# Gender Moderates the Neural Impact of Problematic Media Use on Working Memory in Preschoolers: An fNIRS Study

**DOI:** 10.3390/brainsci15080818

**Published:** 2025-07-30

**Authors:** Keya Ding, Xinyi Dong, Yu Xue, Hui Li

**Affiliations:** 1 Shanghai Institute of Early Childhood Education, Shanghai Normal University, Shanghai 200233, China; keya@shnu.edu.cn (K.D.); 1000462312@smail.shnu.edu.cn (X.D.); 1000530352@smail.shnu.edu.cn (Y.X.); 2 Faculty of Education and Human Development, The Education University of Hong Kong, Hong Kong SAR, China; 3 Macquarie School of Education, Macquarie University, Macquarie Park, NSW 2113, Australia

**Keywords:** fNIRS, problematic media use, working memory, preschool children, gender

## Abstract

Background: This study investigated the relationship between problematic media use (PMU) and working memory in preschoolers. Methods: Parents of children aged 3 to 7 (260 boys, 257 girls; *M_age_* = 5.57, *SD* = 0.73) in Jinan, China, completed questionnaires assessing children’s PMU and working memory. Subsequently, High (*n_high_* = 32, *M_age_* = 4.53, *SD* = 0.67) and Low (*n_low_* = 30, *M_age_* = 4.67, *SD* = 0.66) PMU groups, based on the survey data, complete a dual 1-back task during functional near-infrared spectroscopy (fNIRS) recording. Results: Behavioral accuracy and reaction time showed no significant group differences. However, a significant interaction between the PMU group and gender on prefrontal activation was observed, *F*(1, 60) = 5.88–7.59, *p*s < 0.05, *ηp*^2^ = 0.09–0.12. High-PMU boys exhibited greater left prefrontal activation than low-PMU boys, while low-PMU girls showed greater activation in these same areas compared to low-PMU boys. A three-way interaction of group, task condition, and gender on prefrontal activation was also found, *F*(2, 60) = 5.81–6.42, *p* < 0.01, *ηp*^2^ = 0.10–0.19, suggesting that neural responses varied by task and participant characteristics. Conclusions: These findings indicate that PMU may be associated with altered prefrontal activation during working memory tasks in preschoolers, with gender playing a moderating role.

## 1. Introduction

The pervasiveness of digital media in the lives of young children has sparked growing concern about problematic media use (PMU)—excessive or uncontrolled exposure that can negatively impact their developing minds and bodies [1]. Preschoolers, in a period of rapid brain development, are particularly vulnerable to the potentially negative effects of PMU, with excessive screen time linked to impairments in crucial cognitive skills, including executive functions [2,3,4,5,6,7,8]. Among these, working memory—a cornerstone of learning, reasoning, and problem-solving [5]—is of vital importance for academic and social success. Despite the recognized importance of working memory and the growing prevalence of PMU, research exploring the neural mechanisms underlying this relationship in preschoolers remains scarce, with only limited investigation using neuroimaging techniques, such as functional near-infrared spectroscopy (fNIRS) [9]. This study addressed this critical gap by employing fNIRS to examine the neural correlates of PMU and its impact on working memory in young children, providing critical insights for developmental theory, educational practices, and effective parenting strategies.

### 1.1. Problematic Media Use in Early Childhood: Theoretical Perspectives

Problematic media use (PMU) in early childhood can be understood through the lens of several interconnected theoretical frameworks. Cognitive developmental theory [10,11] emphasizes children’s cognitive immaturity and susceptibility to media influence, which can impact their ability to distinguish between fantasy and reality and potentially hinder opportunities for crucial social interaction. Social cognitive theory [12] emphasizes observational learning and modeling, suggesting that media can shape children’s behaviors and attitudes. Self-determination theory [13] posits that excessive media use may hinder children’s innate psychological needs for autonomy, competence, and relatedness. The displacement hypothesis highlights how media consumption can supplant developmentally vital activities, such as play and social interaction [14]. Uses and gratifications theory [15] considers children’s active choices in media consumption as a means to fulfill specific needs, while Bronfenbrenner’s [16] ecological systems theory emphasizes the interplay of various environmental systems, including family and cultural norms, in shaping media use patterns. These frameworks collectively provide a comprehensive perspective on the complex interplay of developmental, social, and environmental factors that contribute to PMU in early childhood.

In particular, the Interactional Theory of Childhood Problematic Media Use (IT-CPU) provides a valuable framework for understanding this complex phenomenon [1]. This theory posits that PMU emerges and persists due to the interaction of distal, proximal, and maintaining factors. Distal factors, representing broader contextual influences, lay the groundwork for potential PMU. Socioeconomic status (SES), parental education, and the digital environment within the home all contribute to children’s early media habits. Research consistently indicates that children from lower SES backgrounds [17,18] and those experiencing higher levels of household chaos [19,20] exhibit greater screen time, thereby elevating their risk for poor mental health outcomes. Parental modeling also plays a significant role; children of parents with problematic screen use are more likely to develop similar patterns [21], suggesting a learned behavior component.

Proximal factors, operating at a more immediate level, directly influence children’s media use. Children’s temperament, particularly traits like emotional dysregulation and oppositional behavior, can contribute to PMU. Caregivers often utilize digital devices as a calming or behavioral management tool in challenging situations, such as during public outings or when children display negative affect [6,22]. This reinforces the association between media use and emotional regulation, potentially leading to increased reliance on screens. Parental media parenting practices are also crucial. A lack of clear media-use guidelines and structure, coupled with parental media use in the presence of children, can unconsciously encourage excessive screen time [23]. These proximal factors, interacting with distal influences, create a context where PMU can take root.

Maintaining factors perpetuate established patterns of PMU. While not extensively explored in the literature regarding young children, these factors could include the reinforcing nature of digital media content, habit formation, and the potential for withdrawal symptoms when media access is restricted. Further research is needed to fully understand the maintaining factors contributing to PMU in early childhood.

### 1.2. PMU and Working Memory: A Complex Relationship

The relationship between media use and working memory, particularly in young children, is complex and marked by inconsistent findings. While some research suggests potential cognitive benefits associated with specific types of media engagement (e.g., educational apps, interactive games) [24,25], these studies often focus on older children and adolescents, limiting their generalizability to preschoolers. Moreover, a growing body of evidence highlights the detrimental effects of excessive or unregulated screen time, especially smartphone overuse, on working memory and broader cognitive abilities. For instance, studies have linked frequent mobile phone use to decreased accuracy and slower performance on working memory tasks in school-aged children [26,27]. However, other research has found no significant association between mobile phone use and working memory in this age group [28]. These inconsistencies may stem from methodological variations across studies, including differences in sample characteristics, measurement tools, and definitions of PMU. Crucially, few studies have directly examined this relationship in preschoolers, a population particularly vulnerable to the impact of early media exposure due to the rapid development of prefrontal regions crucial for working memory [29].

This study addresses this critical gap by using a combined behavioral and neuroimaging (fNIRS) approach to investigate the association between PMU and working memory performance in preschoolers, aiming to provide a more nuanced understanding of the cognitive consequences of early media exposure in this vulnerable population. In particular, this study seeks to examine the following hypotheses:

**Hypothesis 1.** 
*Preschoolers with higher levels of PMU will exhibit significantly lower behavioral performance on working memory tasks, as measured by accuracy and response time.*

**Hypothesis 2.** 
*Preschoolers with higher levels of PMU will exhibit reduced activation in the prefrontal cortex during working memory tasks, as measured by fNIRS oxygenated hemoglobin changes.*

**Hypothesis 3.** 
*The relationship between PMU and working memory performance will be moderated by individual factors such as gender, exploring potential differential vulnerabilities to the effects of excessive media use.*

This study, by testing these hypotheses, will address a critical gap in the existing literature by providing the first neuroimaging evidence of the relationship between PMU and working memory in preschool children, paving the way for future research in this important area.

## 2. Materials and Methods

### 2.1. Participants

All participants were initially recruited from two private kindergartens in Jinan, Shandong Province, China. A total of 456 parents of children aged 3 to 7 years (238 boys, 218 girls, *M_age_* = 5.57 years, *SD_age_* = 0.74) completed the Problematic Media Use Measure-Short Form (PMUM-SF) questionnaire. Based on the scores of PMUN-SF, the high- and low-problematic media use groups were recruited using the extreme grouping method, resulting in a total of 79 children. All participants were reported to be right-handed, with normal intelligence and no history of neurological disease, loss of consciousness, sensory impairment, autism spectrum disorder, intellectual disability, or learning disabilities. After the start of the experiment, three participants dropped out: one boy from the high-PMU group and two boys from the low-PMU group. An additional 14 participants were excluded due to poor data quality or missing data. Specifically, in the high-PMU group, five girls and two boys were excluded; in the low-PMU group, two girls and five boys were excluded. Poor data quality was determined based on excessive motion artifacts or signal loss in the fNIRS recordings. After these exclusions, the final sample included 62 children, with 32 in the low group (*M_age_* = 4.53 years, *SD_age_* = 0.67) and 30 in the high group (*M_age_* = 4.67 years, *SD_age_* = 0.66). The low PMU group consisted of 21 girls and 11 boys, while the high PMU group included 16 girls and 14 boys. A priori power analysis using G*Power 3.1 [30], assuming a medium effect size (ƒ = 0.25), an alpha level of 0.05, and a power of 0.85, indicated a minimum sample size of 44 participants. So, the sample size is adequate for this study. The Research Ethics Committee of Shanghai Normal University approved the study. Written informed consent was obtained from all parents, kindergarten principals, and educational directors (class teachers) prior to participation. All questionnaires were completed by parents between November and December 2023. Subsequently, the fNIRS data were collected in December 2023. Participants were informed of the study’s voluntary nature and their right to withdraw at any time. All participants received a toy as a reward for participating in the study.

### 2.2. Measures

Demographic Questionnaire: An adapted demographic questionnaire collected information on region, child’s age and gender, number of adults and children in the household, parents’ education levels, occupations, and annual household income [17]. Parental education was categorized into six levels: “Primary school or below,” “Middle school,” “High school or vocational school,” “Junior college,” “Bachelor’s degree,” and “Postgraduate or above,” and coded from 1 to 6, with higher scores indicating higher educational attainment. Parental occupations were coded on a 1–10 scale, with higher scores indicating higher occupational status. SES was computed by summing the standardized z-scores of parental education and occupation scores [31,32]. Higher composite scores indicate higher socioeconomic status.

Combined Raven’s Test (CRT): The CRT was administered to all participants to assess individual and group differences in intelligence quotient (IQ). Li et al. [33] adapted the test and validated it in a sample from Shanghai (a city in China). The findings demonstrated that the CRT aligns well with the cognitive development levels of Chinese children, exhibiting high reliability and moderate validity. Given the potential upward trend in children’s IQ scores over the past few decades, this study utilized raw scores for statistical analyses [34].

Problematic Media Use Measure-Short Form (PMUM-SF): The PMUM-SF was developed to assess problematic media use (PMU) in young children. The Chinese version of the PMUM has been validated for use in Chinese preschoolers [35]. This PMUM-SF consisted of 9 items (e.g., “It is hard for my child to stop using screen media”), which required parents to rate their children’s frequency of daily media use on a 5-point Likert scale, ranging from “never” to “always” (1 = *Never*, 5 = *Always*). The final score of PMU was calculated by summing the scores of each item. The reliability of this scale, as measured by Cronbach’s α, was found to be 0.93.

**Figure 1:**
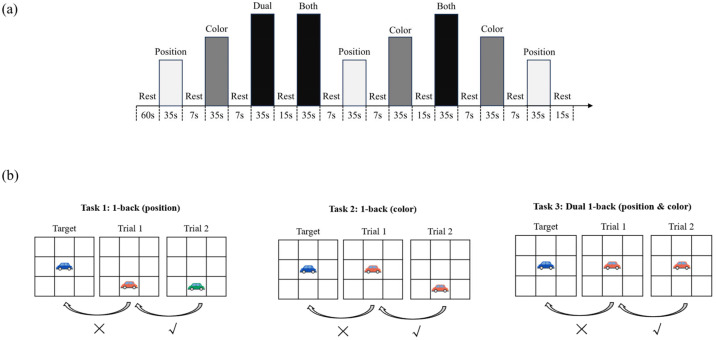
Block design and example trial of the Dual 1-back working memory task: (**a**) The experimental task comprised three conditions: (1) Position-only 1-back, where children judged whether the car’s position matched the previous trial. (2) Color-only 1-back, where they judged whether the car’s primary color matched. (3) Dual 1-back, where both position and color had to match simultaneously. (**b**) One sample trial of the Dual 1-back condition. Within each trial, a cartoon car stimulus was shown, and children responded by pressing the “✓” key for a match and the “×” key for a non-match.

Dual 1-back Task: Based on the dual n-back paradigm [36] and the visuospatial n-back Task adapted for children [37], this study employed a modified dual 1-back task to assess the visuospatial working memory of preschool children aged 4–6. To enhance engagement and child-friendliness, we redesigned the stimuli using cartoon vehicles with bright colors, replacing the standard geometric shapes typically used in previous versions. The Task required participants to track both the position and color of the stimulus (a cartoon car) on a 3 × 3 grid displayed at the center of a gray computer screen. The working memory task consisted of three experimental conditions designed to tap into different cognitive demands: (1) Position-only 1-back, in which children were asked to judge whether the car appeared in the same spatial location as in the previous trial; (2) Color-only 1-back, where they judged whether the car’s primary color matched that of the preceding trial; and (3) Dual 1-back, where both spatial position and color had to match simultaneously. Each condition was repeated in three blocks, presented in a pseudo-randomized order, resulting in a total of nine blocks and 81 trials across the task. (see Figure 1a). Within each block, nine stimulus trials were presented, with each trial containing one matching stimulus and six non-matching ones (i.e., match:non-match ratio = 3:6 + 1). Before the formal experiment began, a guided practice session was conducted. Children received auditory feedback during the practice: a cheerful “Oh-ho!” sound for correct responses and a neutral “beep” for errors or missing responses. Once the child achieved an accuracy rate above 80%, the formal experiment began without auditory feedback.

**Figure 2:**
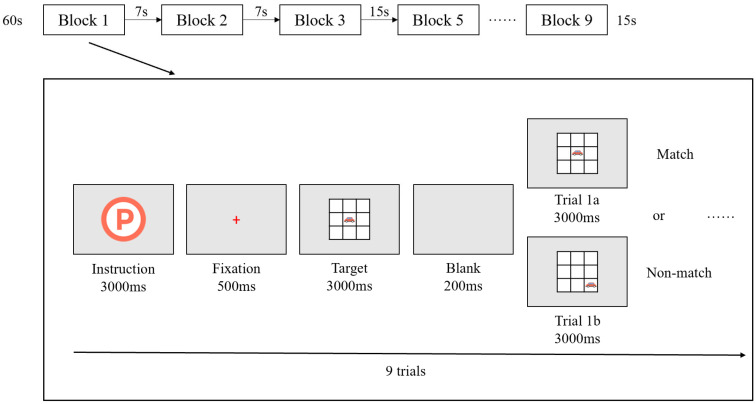
The working memory task of the Dual 1-back.

The Task was programmed and presented using E-Prime 3.0 (Psychology Software Tools, Inc., Pittsburgh, PA, USA), and all stimuli were displayed on a 16-inch laptop screen with a resolution of 1024 × 768 pixels. Each block began with a 300 ms task instruction screen indicating the type of upcoming condition, followed by a red fixation cross displayed for 500 ms. Then, the target stimulus (a cartoon car) appeared on the screen for 3000 ms. During each trial, participants had to respond to whether the current stimulus matched the one presented in the previous trial based on the task condition. As shown in Figure 1b, they need to respond by pressing either the “✓” key (match) or the “×” key (non-match). If no response was made within 5000 ms, the stimulus disappeared automatically. A 200 ms blank screen followed each trial before the next stimulus appeared. The entire Task lasted approximately 7–8 min (Figure 2).

### 2.3. fNIRS Data Recording and Processing

A portable fNIRS device (NirSport2, NIRx Medizintechnik GmbH, Germany) was used to collect brain imaging data from children. The system employed wavelengths of 760 and 850 nm, with a sampling frequency of 5.1 Hz, and operated using the Aurora fNIRS 2021.9 acquisition software. According to the international 10-10 transcranial localization system, 16 sources and 16 detectors were arranged to mainly cover bilateral prefrontal regions, including the inferior frontal gyrus (IFG), middle frontal gyrus (MFG), and superior frontal gyrus (SFG), with a standard inter-optode distance of 3 cm. To minimize external environmental interference on children’s performance, the fNIRS experiment was conducted in a sound-attenuated meeting room within the kindergarten. This room, typically used by staff for meetings, was physically separated from the classrooms and outdoor activity areas, providing an acoustically quiet environment for testing.

**Figure 3:**
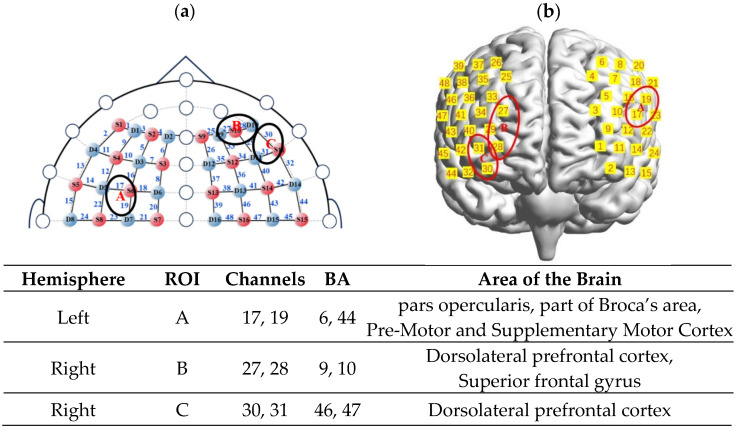
fNIRS probe configuration and the specific information of ROIs: (**a**) 2D schematic of the fNIRS probe placement, showing the source-detector arrangement and corresponding measurement channels; (**b**) 3D reconstruction of the probe layout and the spatial localization of regions of interest (ROIs) projected onto cortical surface.

After data collection, a probabilistic registration method was applied in MATLAB to map the channel positions to MNI space. Corresponding Brodmann areas (BA) are listed in Table A1. Based on prior fNIRS research in preschoolers [38,39], the 48 channels were grouped into three regions of interest (ROIs): ROI-A included channels 17 and 19, corresponding to BAs 6 and 44; ROI-B included channels 27 and 28, corresponding to BAs 9 and 10; and ROI-C included channels 30 and 31, corresponding to BAs 46 and 47 (Figure 3).

Imaging data preprocessing was performed using MATLAB R2017b. The Homer2 NIRS toolbox [40] was employed to apply discrete wavelet transform for motion artifact removal, followed by bandpass filtering (0.01–0.2 Hz) to reduce low-frequency drifts and high-frequency physiological noise [41]. Subsequently, raw optical signals were converted into hemoglobin concentration changes using the modified Beer-Lambert law [41]. Considering that oxygenated hemoglobin (HbO) is the most sensitive indicator of local cerebral blood flow changes [42,43], average HbO concentration changes in each channel were extracted and exported for further analysis. Due to version-specific updates in certain built-in functions required for artifact screening and signal filtering, we used R2017b for initial preprocessing steps and R2021b for visualization.

### 2.4. Statistical Analysis

Behavioral data (accuracy and reaction time) from the dual 1-back working memory task were processed and analyzed using IBM SPSS Statistics 27.0. Accuracy and reaction time data were initially exported from E-Prime 3.0 and merged using E-Merge 3. Invalid trials were removed prior to analysis. To examine the effects of problematic media use (PMU), gender, and stimulus condition on task performance, a 2 (PMU Group: high vs. low) × 2 (Gender: boys vs. girls) × 3 (Stimulus Condition: position, color, dual) repeated measures ANOVA was conducted. Given our *a priori* hypotheses regarding specific ROIs, separate repeated-measures ANOVAs were then conducted for each ROI (BA6, BA9, and BA44) to examine the effects of PMU, gender, and stimulus condition on brain activation (oxygenated hemoglobin). The False Discovery Rate (FDR) correction was applied to control for multiple comparisons across the three ROIs for both behavioral and fNIRS analyses.

## 3. Results

### 3.1. Behavioral Results

#### 3.1.1. Preliminary Analyses

To ensure groups were comparable on background characteristics, independent samples *t*-tests were conducted for continuous variables (age, Standard Progressive Matrices [SPM] score, parents’ education level, parents’ occupation, annual family income, and socioeconomic status [SES]), and a chi-square test was conducted for the categorical variable of gender. As shown in Table 1, there were no significant differences between the high and low PMU groups on any of these demographic variables.

#### 3.1.2. Behavioral Task Performance

Descriptive statistics for accuracy and reaction time (RT) on the dual 1-back task, broken down by PMU group and gender, are presented in Table 2. A 2 (PMU Group: high vs. low) × 2 (Gender: boys vs. girls) × 3 (Stimulus Condition: position, color, dual) repeated measures ANOVA was conducted to analyze task performance.

Accuracy. The analysis revealed a significant main effect of Stimulus Condition, *F*(2, 122) = 8.27, *p* = 0.001, $\eta_{p}^{2}$ = 0.12. Accuracy was significantly lower in the position condition (*M* = 0.60, *SD* = 0.17) compared to both the color (*M* = 0.69, *SD* = 0.16, *p* = 0.001) and dual-task conditions (*M* = 0.66, *SD* = 0.15, *p* = 0.011). Neither the main effects of PMU Group, *F*(1, 61) = 1.56, *p* = 0.217, $\eta_{p}^{2}$ = 0.03, and Gender, *F*(1, 61) = 2.65, *p* = 0.109, $\eta_{p}^{2}$ = 0.04, nor any of the interaction effects reached statistical significance (all *p*s > 0.05). Although the main effect of PMU Group was not significant, children in the high PMU group tended to exhibit numerically lower accuracy across all task conditions compared to the low PMU group, a trend that was more pronounced among boys.

Reaction Time. The repeated measures ANOVA on RT revealed no significant main effects of PMU Group, *F*(1, 61) = 0.03, *p* = 0.859, $\eta_{p}^{2}$ < 0.01; Gender, *F*(1, 61) = 2.70, *p* = 0.106, $\eta_{p}^{2}$ = 0.04; or Stimulus Condition, *F*(2, 122) = 0.24, *p* = 0.787, $\eta_{p}^{2}$ < 0.01. None of the interaction effects were significant (all *p*s > 0.05; see Table 3). Therefore, Hypothesis 1, which predicted lower behavioral performance in the high PMU group, was not supported.

### 3.2. fNIRS Results

#### 3.2.1. Differences in Brain Activation Between PMU Groups

The repeated measures *ANOVA* was conducted to compare brain activation levels between children with high and low levels of PMU. The results showed no significant differences between the two groups in all regions of interest (ROIs). Specifically, no significant group differences were found in ROI A (*F* = 0.48, *p* = 0.490, $\eta_{p}^{2}$ = 0.01), ROI B (*F* = 0.01, *p* = 0.934, $\eta_{p}^{2}$ = 0.00), and ROI C (*F* = 0.04, *p* = 0.848, $\eta_{p}^{2}$ = 0.00).

#### 3.2.2. Differences in Brain Activation Between Genders

To examine potential gender differences in brain activation during the working memory task, a repeated measures *ANOVA* was performed. The results showed no significant differences between boys and girls across all ROIs. Specifically, no gender differences were found in ROI A (*F* = 0.19, *p* = 0.662, $\eta_{p}^{2}$ = 0.00), ROI B (*F* = 0.44, *p* = 0.510, $\eta_{p}^{2}$ = 0.01), and ROI C (*F* = 0.05, *p* = 0.822, $\eta_{p}^{2}$ = 0.00).

#### 3.2.3. Differences in Brain Activation Under Different Stimulus Conditions

A repeated measures *ANOVA* was conducted to investigate the effects of different stimulus conditions (position, color, and dual-task) on brain activation. The results revealed no significant main effects of stimulus condition on brain activation in any ROI. Specifically, ROI A (*F* = 2.39, *p* = 0.096, $\eta_{p}^{2}$ = 0.04), ROI B (*F* = 0.99, *p* = 0.372, $\eta_{p}^{2}$ = 0.02), and ROI C (*F* = 0.34, *p* = 0.712, $\eta_{p}^{2}$ = 0.01) showed no significant activation differences under different task conditions.

#### 3.2.4. Brain Activation Differences Under the Interaction Between Group and Gender

**Figure 4:**
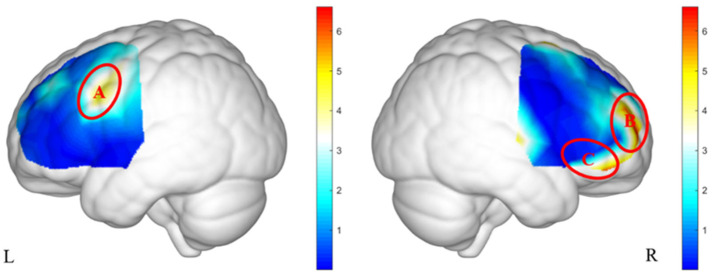
The brain activation heatmaps under the interaction of group and gender.

As shown in Figure 4, significant interaction effects between group and gender were observed in ROI A (*F*(61) = 5.88, *p* = 0.027, $\eta_{p}^{2}$ = 0.09), ROI B (*F*(61) = 7.59, *p* = 0.024, $\eta_{p}^{2}$ = 0.12), and ROI C (*F*(61) = 4.67, *p* = 0.035, $\eta_{p}^{2}$ = 0.08).

Post hoc analyses (see Table 4) revealed that in ROI A, boys in the high PMU group exhibited significantly higher brain activation than boys in the low PMU group (*p* = 0.048). In contrast, in the low PMU group, girls showed significantly greater activation than boys (*p* = 0.049). In ROI B, girls in the high PMU group showed higher activation compared to girls in the low PMU group (*p* = 0.029), whereas in the low PMU group, boys exhibited greater activation than girls (*p* = 0.020). No significant post hoc differences were observed in ROI C.

#### 3.2.5. Interaction Between Stimulus Condition and Gender

The interaction effects between stimulus condition and gender were not statistically significant in any of the regions of interest, including ROI A (*F*(61) = 0.83, *p* = 0.439, $\eta_{p}^{2}$ = 0.01), ROI B (*F*(61) = 2.64, *p* = 0.078, $\eta_{p}^{2}$ = 0.04), and ROI C (*F*(61) = 0.55, *p* = 0.577, $\eta_{p}^{2}$ = 0.01). These results suggest that the influence of stimulus conditions on brain activation did not vary significantly by gender.

#### 3.2.6. Three-Way Interaction of PMU Group, Stimulus Condition, and Gender

**Figure 5:**
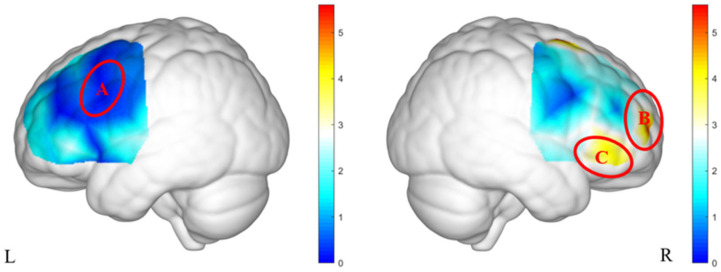
The brain activation heatmaps under the three-way interaction of the group, stimulus condition, and gender.

Further analysis revealed significant three-way interaction effects among the group, stimulus condition, and gender in ROI B (*F*(61) = 6.42, *p* = 0.006, $\eta_{p}^{2}$ = 0.10) and ROI C (*F*(61) = 5.81, *p* = 0.006, $\eta_{p}^{2}$ = 0.19), as presented in Figure 5.

Post hoc analyses revealed several three-way interactions between sex, PMU group (low vs. high), and task condition within specific regions of interest (ROIs; see Table 5). In ROI B, during the color task, boys in the low PMU group exhibited significantly greater activation than girls in the low PMU group (*p* = 0.001). Boys in the low PMU group also showed greater activation during the color task than the position task (*p* = 0.039). Boys in the high PMU group demonstrated significantly greater activation during the dual-task condition compared to the color task (*p* = 0.025). In ROI C, a significant interaction was observed such that boys in the high PMU group exhibited greater activation during the dual-task condition compared to boys in the low PMU group (*p* = 0.009), whereas girls in the high PMU group showed significantly less activation during the dual-task condition compared to girls in the low PMU group (*p* = 0.027). Furthermore, within the low PMU group, girls demonstrated greater activation during the dual-task condition compared to boys (*p* = 0.021). Conversely, within the high PMU group, boys exhibited significantly greater activation during the dual-task condition than girls (*p* = 0.012).

## 4. Discussion

This study investigated the relationship between problematic media use (PMU) and working memory in preschoolers, examining both behavioral performance and neural activation patterns. While behavioral performance did not significantly differ across PMU levels, fNIRS revealed distinct patterns of neural engagement, moderated by gender. These findings contribute to understanding the potential neural mechanisms by which early media exposure may influence executive functioning.

### 4.1. Working Memory Performance and Problematic Media Use

Although preschoolers with higher levels of PMU exhibited a trend toward lower accuracy on working memory tasks compared to their low-PMU peers, this difference did not reach statistical significance. This null finding at the behavioral level requires careful consideration. One possibility is that the sample size, while adequate for detecting neural effects, may have lacked the power to reveal subtle behavioral differences. Another explanation could be that the behavioral measures employed were not sensitive enough to capture the specific working memory processes affected by PMU. It is also plausible that the observed neural changes reflect compensatory mechanisms that allow children with high PMU to maintain behavioral performance despite underlying neural inefficiencies. This interpretation aligns with the neural compensation hypothesis [44,45], which posits that alternate neural pathways are recruited to maintain performance when primary cognitive circuits are compromised. These neural alterations may not reflect isolated impairments in working memory, but rather stem from a broader disruption in attentional regulation. Recent neuroimaging research on short video addiction (SVA) has shown that individuals with high SVA levels exhibit increased regional homogeneity in the thalamus, a finding interpreted as enhanced sensory sensitivity coupled with diminished attentional control [46]. This pattern of over-activation is thought to reinforce compulsive engagement with highly stimulating digital content, such as short videos. In this light, PMU may disrupt children’s capacity to filter and prioritize task-relevant information, thereby undermining working memory through impaired attentional disengagement.

The absence of significant gender differences in working memory performance between PMU groups was unexpected. This may be attributed to the relative homogeneity of the sample regarding distal (e.g., socioeconomic status, digital environment), proximal (e.g., parenting style), and maintaining factors (e.g., parental regulation strategies) proposed by the IT-CPU [1]. These contextual factors may have moderated or buffered gender-related behavioral differences in this cohort.

### 4.2. Gender-Specific Brain Activation Patterns

While behavioral differences were non-significant, fNIRS data revealed distinct neural activation patterns. Girls exhibited greater activation in left-hemisphere regions associated with language processing (Broca’s area, premotor cortex, supplementary motor area [BA6], inferior frontal gyrus [BA44]), while boys showed stronger activation in the right dorsolateral prefrontal cortex (DLPFC; BA9 and BA10), associated with spatial processing and executive functions [47,48]. These findings suggest that even in preschool, gender-specific neural pathways for cognitive processing are evident, potentially reflecting a preference for verbal encoding in girls and visuospatial strategies in boys, consistent with Dual-Coding Theory [49].

The significant interaction between gender and PMU on brain activation further supports the moderating role of gender. Boys with high PMU showed increased activation in the same left-hemisphere language areas preferentially activated in girls with low PMU. Conversely, girls with high PMU exhibited increased activation in the right DLPFC, typically more active in boys with low PMU. These findings suggest compensatory neural recruitment in response to cognitive demands that are overloaded by excessive media exposure. Boys may shift away from their typical visuospatial dominance toward language-related areas, while girls may engage more in domain-general executive regions. Nevertheless, we need more empirical evidence to verify this compensatory neural recruitment mechanism.

### 4.3. Three-Way Interaction Effects

It is interesting to note that the three-way interaction of group, condition, and gender on DLPFC activation (ROI B and C) provides further nuance. In ROI B, boys with low PMU showed higher activation during the color task than girls and during the color task compared to the position task. Boys with high PMU showed higher activation during the dual-task than during the color task. In ROI C, boys with high PMU exhibited higher activation during the dual Task than those with low PMU, while girls showed the opposite pattern. These findings suggest that PMU’s impact on neural function is context-specific, varying depending on the cognitive demands of the Task and the individual’s gender. These complex interactions warrant further investigation to understand the precise mechanisms by which PMU affects specific working memory processes and, ultimately, to elucidate the sophisticated relationships between task, gender, and working memory.

In conclusion, this study provides emerging evidence that excessive media exposure in early childhood is associated with altered neural engagement during working memory tasks and that these effects are moderated by gender. These results underscore the need for tailored intervention strategies that account for gender differences in cognitive processing styles and neural plasticity. Future longitudinal studies are warranted to track whether these compensatory patterns persist, intensify, or diminish over time and to investigate their relationship with long-term cognitive and emotional development.

## 5. Conclusions, Limitations, and Implications 

In conclusion, this study provides novel insights into the relationship between problematic media use (PMU) and working memory in preschool-aged children. While behavioral performance on the dual 1-back task did not differ significantly between high and low PMU groups, neuroimaging data revealed distinct patterns of prefrontal activation associated with PMU levels and gender. Specifically, high-PMU boys and low-PMU girls exhibited heightened activation in the left prefrontal regions, suggesting that PMU may differentially influence neural functioning across genders. Moreover, the observed three-way interaction among the PMU group, task condition, and gender underscores the context-dependent nature of these neural effects. These findings highlight the importance of considering both individual and situational factors when examining the cognitive and neural implications of early media exposure. Future research should further investigate the developmental trajectories and long-term consequences of PMU on executive functioning, ideally incorporating longitudinal designs and a range of diverse cognitive tasks.

This study has limitations, including reliance on parent-reported PMU, lack of control for pre-existing cognitive differences and socioeconomic status, and the limited spatial resolution of fNIRS, which restricts our ability to localize activation precisely or to observe deeper cortical and subcortical structures. In addition, the current analysis only examined unilateral (left-hemisphere) ROIs, and this approach may have overlooked potential bilateral or right-hemisphere effects. Future studies should adopt bilateral ROI analysis and broader cortical coverage to improve interpretability. Future research should address these limitations by incorporating objective measures of media use, controlling for relevant covariates, and using larger and more diverse samples. Longitudinal studies are crucial to track the developmental trajectory of these neural patterns and their long-term cognitive and emotional consequences. Furthermore, research exploring the effectiveness of interventions designed to mitigate the adverse effects of PMU on working memory, tailored to address gender-specific neural vulnerabilities, is warranted.

Despite these limitations, the findings offer important implications for early childhood education and parenting practices. First, the study suggests that excessive media use in early childhood may be associated with altered neural processing during working memory tasks, although no behavioral differences were observed. While these findings raise questions about the potential cognitive risks, particularly in the domain of working memory, further research is needed to determine whether these neural differences translate into functional impairments or represent transient compensatory mechanisms. This highlights the importance of collaborative efforts between educators and parents in establishing healthy media habits and minimizing excessive screen time. Second, the observed gender-specific neural responses underscore the need for tailored educational strategies that take into account individual differences. Third, this research contributes to the growing body of literature utilizing neuroimaging techniques in early childhood education, providing biological evidence to inform media usage guidelines and cognitive development strategies. By integrating these findings into educational practice, we can better support children’s cognitive development in the digital age.

## Figures and Tables

**Table 1:** Demographic characteristics of participants.

Variable	Low PMU Group (*n* = 32)	High PMU Group (*n* = 30)	*F*	*p*	$\eta_{p}^{2}$
	*M* (*SD*)	*M* (*SD*)			
Gender ^1^	11/21	14/16	0.972	0.438	-
Age	4.67 (0.66)	4.53 (0.67)	0.640	0.427	0.011
SPM ^2^	22.56 (9.68)	21.87 (6.01)	0.114	0.737	0.002
Father’s education level	5.00 (0.14)	5.03 (0.14)	0.033	0.864	0.000
Mother’s education level	4.88 (0.79)	5.17 (0.59)	1.317	2.664	0.108
Father’s occupation	3.63 (1.54)	3.50 (1.98)	0.078	0.781	0.001
Mother’s occupation	5.03 (2.60)	5.37 (2.39)	0.280	0.599	0.000
Annual family income	8.13 (2.35)	8.03 (2.53)	0.022	0.883	0.000
SES	−0.25 (2.00)	0.27 (1.67)	1.210	0.276	0.020

^1^ The number of participants by gender is reported and analyzed using the chi-square test; ^2^ SPM refers to the raw score of the Combined Urban Standard Progressive Matrices (Raven’s test).

**Table 2:** ACC and RT in the Dual 1-back working memory task across PMU groups and gender.

Variable	Low-PMU Girls (*n* = 21)	Low-PMU Boys (*n* = 11)	High-PMU Girls (*n* = 16)	High-PMU Boys (*n* = 14)	All (*n* = 63)	*F*	*p*
	*M* ± *SD*	*M* ± *SD*	*M* ± *SD*	*M* ± *SD*	*M* ± *SD*		
ACC-P	0.64 ± 0.18	0.62 ± 0.13	0.58 ± 0.20	0.56 ± 0.15	0.60 ± 0.17	0.004	0.953
ACC-C	0.74 ± 0.17	0.68 ± 0.14	0.71 ± 0.18	0.61 ± 0.13	0.69 ± 0.16		
ACC-D	0.71 ± 0.17	0.62 ± 0.12	0.68 ± 0.17	0.62 ± 0.11	0.66 ± 0.15		
RT-P	1339.46 ± 215.90	1265.97 ± 191.90	1350.52 ± 232.88	1259.32 ± 202.99	1311.18 ± 212.38	0.866	0.356
RT-C	1312.80 ± 290.00	1222.65 ± 204.17	1283.10 ± 179.03	1288.05 ± 225.22	1283.55 ± 232.52		
RT-D	1368.52 ± 225.54	1197.93 ± 332.11	1289.43 ± 299.30	1283.18 ± 271.15	1298.57 ± 275.76		

Note. ACC = accuracy, RT = reaction time, P = position, C = Color, D = Dual.

**Table 3:** Accuracy and RT in the Dual 1-back Task across PMU groups and gender.

	ACC			RT		
	*F*	*p*	$\eta_{p}^{2}$	*F*	*p*	$\eta_{p}^{2}$
Group	1.561	0.217	0.026	0.032	0.859	0.011
Gender	2.652	0.109	0.044	2.699	0.106	0.044
Stimulus Condition	8.268	0.001	0.125	0.240	0.787	0.004
Group × Gender	0.004	0.953	0.000	0.866	0.356	0.015
Stimulus Condition × Group	0.765	0.468	0.013	0.024	0.976	0.000
Stimulus Condition × Gender	1.110	0.333	0.019	0.191	0.826	0.003
Stimulus Condition × Group × Gender	0.331	0.719	0.006	0.651	0.523	0.011

Note. ACC = accuracy, RT = reaction time.

**Table 4:** Differences in brain activation under the interaction of group and gender.

ROI	Interaction of Group and Gender			
	*F*	*p*	$\eta_{p}^{2}$	Post Hoc
A	5.88	0.027	0.09	Boys in High PMU group > Boys in Low PMU group; Girls > Boys in Low PMU group
B	7.59	0.024	0.12	Girls in High PMU group > Girls in Low PMU group; Boys > Girls in Low PMU group
C	4.67	0.035	0.08	No significant differences

**Table 5:** Differences in brain activation: three-way interaction.

ROI	Interaction	*F*	*p*	$\eta_{p}^{2}$	Post Hoc
B	Group × Condition × Gender	6.42	0.006	0.10	Low PMU, Color: Boys > Girls; Low PMU, Boys: Color > Position; High PMU, Boys: Dual > Color
C	Group × Condition × Gender	5.81	0.006	0.19	Low PMU, Dual: Girls > Boys; High PMU, Dual: Boys > Girls; Dual, Boys: High PMU > Low PMU; Dual, Girls: Low PMU > High PMU

## Data Availability

We are committed to sharing our data openly and transparently. The dataset of the present study, including deidentified participant data, processed brain measures, and functional assessments, will be made available upon reasonable request. Requests may be made to the corresponding authors, and approval from the sponsoring institution (Shanghai Normal University) is required.

## Abbreviations

The following abbreviations are used in this manuscript:

PMU	Problematic Media Use
fNIRS	functional Near-Infrared Spectroscopy
IT-CPU	Interactional Theory of Childhood Problematic Media Use
SES	Socioeconomic Status
P.R.C	People’s Republic of China
PMUM-SF	Problematic Media Use Measure-Short Form
CRT	Combined Raven’s Test
IFG	Inferior Frontal Gyrus
MFG	Middle Frontal Gyrus
SFG	Superior Frontal Gyrus
MNI	Montreal Neurological Institute
BA	Brodmann Areas
ROI	Regions of Interest

## Appendix A

**Table A1:** Brodmann areas corresponding to fNIRS channels.

Channels	BA	Brain Coverage (%)
CH1	10—Frontopolar area	38.6%
	46—Dorsolateral prefrontal cortex	35.7%
CH2	46—Dorsolateral prefrontal cortex	51.9%
	47—Inferior prefrontal gyrus	47.3%
CH3	10—Frontopolar area	84.5%
CH4	10—Frontopolar area	99.3%
CH5	46—Dorsolateral prefrontal cortex	87.6%
CH6	9—Dorsolateral prefrontal cortex	51.6%
CH7	9—Dorsolateral prefrontal cortex	48.0%
	46—Dorsolateral prefrontal cortex	52.0%
CH8	8—Includes Frontal eye fields	55.5%
	9—Dorsolateral prefrontal cortex	44.5%
CH9	46—Dorsolateral prefrontal cortex	83.7%
CH10	45—pars triangularis Broca’s area	70.6%
CH11	45—pars triangularis Broca’s area	54.5%
	46—Dorsolateral prefrontal cortex	36.5%
CH12	45—pars triangularis Broca’s area	94.4%
CH13	38—Temporopolar area	100.0%
CH14	48—Retrosubicular area	59.9%
CH15	21—Middle Temporal gyrus	100.0%
CH16	45—pars triangularis Broca’s area	51.7%
CH17	44—pars opercularis, part of Broca’s area	75.4%
CH18	9—Dorsolateral prefrontal cortex	79.4%
CH19	6—Pre-Motor and Supplementary Motor Cortex	92.5%
CH20	6—Pre-Motor and Supplementary Motor Cortex	77.2%
CH21	4—Primary Motor Cortex	60.7%
CH22	43—Subcentral area	68.7%
CH23	1—Primary Somatosensory Cortex	34.4%
CH24	10—Frontopolar area	100.0%
CH25	10—Frontopolar area	100.0%
CH26	9—Dorsolateral prefrontal cortex	52.8%
	10—Frontopolar area	36.8%
CH27	10—Frontopolar area	81.1%
CH28	10—Frontopolar area	38.2%
CH29	46—Dorsolateral prefrontal cortex	75.6%
CH30	46—Dorsolateral prefrontal cortex	47.7%
	47—Inferior prefrontal gyrus	52.3%
CH31	45—pars triangularis Broca’s area	46.2%
	46—Dorsolateral prefrontal cortex	47.8%
CH32	38—Temporopolar area	96.0%
CH33	46—Dorsolateral prefrontal cortex	76.1%
CH34	45—pars triangularis Broca’s area	57.1%
	46—Dorsolateral prefrontal cortex	42.9%
CH35	9—Dorsolateral prefrontal cortex	63.8%
	46—Dorsolateral prefrontal cortex	36.2%
CH36	45—pars triangularis Broca’s area	41.9%
CH37	8—Includes Frontal eye fields	60.2%
	9—Dorsolateral prefrontal cortex	39.8%
CH38	9—Dorsolateral prefrontal cortex	77.8%
CH39	6—Pre-Motor and Supplementary Motor Cortex	75.4%
CH40	45—pars triangularis Broca’s area	100.0%
CH41	44—pars opercularis, part of Broca’s area	78.4%
CH42	48—Retrosubicular area	51.0%
CH43	6—Pre-Motor and Supplementary Motor Cortex	32.3%
	43—Subcentral area	63.6%
CH44	21—Middle Temporal gyrus	100.0%
CH45	21—Middle Temporal gyrus	33.0%
	22—Superior Temporal Gyrus	67.0%
CH46	6—Pre-Motor and Supplementary Motor Corte	94.3%
CH47	1—Primary Somatosensory Cortex	35.1%
CH48	4—Primary Motor Cortex	57.6%
	6—Pre-Motor and Supplementary Motor Cortex	34.5%
